# Validating Ligands with Phenix

**DOI:** 10.1063/4.0001014

**Published:** 2025-10-27

**Authors:** Dorothee Liebschner

**Affiliations:** Lawrence Berkeley National Laboratory, Berkeley, CA, USA

## Abstract

Accurate modeling of ligands in macromolecular structures is essential for structure-based drug discovery. X-ray crystallography can provide such accurate models of protein-ligand complexes (1, 2). Since ligands typically contribute only a small portion of the total scattering signal and may bind with partial occupancy, their presence must be rigorously validated (3). Traditionally, ligand validation has relied on model-to-data fit metrics, such as real-space correlation to electron density maps in crystallography (Fig. 1). While such metrics remain important (4, 5), additional validation criteria can provide a more holistic assessment. This presentation will highlight a new tool in Phenix that evaluates ligand quality in crystallographic models using a combination of model-to-data fit, geometric parameters, stereochemistry, B-factors, hydrogen bonding, and steric clash analysis. We will demonstrate how these validation metrics can help identify modeling errors and increase confidence in ligand interpretation.

